# Proanthocyanidins Alleviates AflatoxinB_1_-Induced Oxidative Stress and Apoptosis through Mitochondrial Pathway in the Bursa of Fabricius of Broilers

**DOI:** 10.3390/toxins11030157

**Published:** 2019-03-10

**Authors:** Shahid Ali Rajput, Cong Zhang, Yue Feng, Xiao Tian Wei, Mahmoud Mohamed Khalil, Imran Rashid Rajput, Dost Muhammad Baloch, Aftab Shaukat, Nasir Rajput, Hammad Qamar, Mubashar Hassan, Desheng Qi

**Affiliations:** 1 Department of Animal Nutrition and Feed Science, College of Animal Science and Technology, Huazhong Agricultural University, Wuhan 430070, China; dr.shahidali@hotmail.com (S.A.R.); zhangcong@webmail.hzau.edu.cn (C.Z.); fengyueyue@webmail.hzau.edu.cn (Y.F.); weixiaotian@webmail.hzau.edu.cn (X.T.W.); 2 Animal Production Department, Faculty of Agriculture, Benha University, 13736 Banha, Egypt; mahmoud.khalil@fagr.bu.edu.eg; 3 Faculty of Veterinary and Animal Science, Department of Biotechnology, Lasbela Univesity of Agriculture Water and Marine Science, 89250 Uthal, Balochistan, Pakistan; drimranrajput@gmail.com (I.R.R.); vc@luawms.edu.pk (D.M.B.); 4 College of Veterinary Medicine, Huazhong Agricultural University, Wuhan 430070, China; aftab.shaukat@webmail.hzau.edu.cn; 5 Department of Poultry Husbandry, Faculty of Animal Husbandry and Veterinary Science, Sindh Agriculture University, 70060 Tandojam, Pakistan; nasirrajput@sau.edu.pk; 6 Research Center of Animal Nutrition and Metabolic Diseases, College of Veterinary Medicine, Huazhong Agricultural University, Wuhan 430070, China; hammadqamar@gmail.com; 7 State Key Laboratory of Agricultural Microbiology, College of Veterinary Medicine, Huazhong Agricultural University, Wuhan 430070, China; mubashar.hassan@webmail.hzau.edu.cn

**Keywords:** aflatoxin B_1_, proanthocyanidins, oxidative stress, apoptosis, bursa of Fabricius, broiler

## Abstract

Aflatoxin B_1_ (AFB_1_) is a serious threat to the poultry industry. Proanthocyanidins (PCs) demonstrates a broad range of biological, pharmacological, therapeutic, and chemoprotective properties. The aim of this study was to investigate the ameliorative effects of PCs against AFB_1_-induced histopathology, oxidative stress, and apoptosis via the mitochondrial pathway in the bursa of Fabricius (BF) of broilers. One hundred forty-four one-day old Cobb chicks were randomly assigned into four treatment groups of six replicates (6 birds each replicate) for 28 days. Groups were fed on the following four diets; (1) Basal diet without addition of PCs or AFB_1_ (Control); (2) basal diet supplemented with 1 mg/kg AFB_1_ from contaminated corn (AFB_1_); (3) basal diet supplemented with 250 mg/kg PCs (PCs); and (4) basal diet supplemented with 1 mg/kg AFB_1_ + 250 mg/kg PCs (AFB_1_+ PCs). The present study results showed that antioxidant enzymes activities of total superoxide dismutase (T-SOD), catalase (CAT), glutathione peroxidase (GSH-Px), and glutathione S-transferase (GST) in AFB_1_ treated group were (*p* < 0.05) decreased, whereas malondialdehyde (MDA) contents were significantly increased in comparison with the control group. Furthermore, we found that dietary PCs treatment ameliorated AFB_1_-induced oxidative stress in the BF through inhibiting the accumulation of MDA content and enhancing the antioxidant enzymes activities (T-SOD, CAT, GSH-Px, and GST). Similarly, PCs markedly enhanced messenger RNA (mRNA) expression of antioxidant genes (*SOD*, *CAT*, *GPx1*, and *GST*) in comparison with AFB_1_ group. Moreover, histological results showed that PCs alleviated AFB_1_-induced apoptotic cells in the BF of broilers. In addition, both mRNA and protein expression results manifested that mitochondrial-apoptosis-associated genes (*Bax*, *caspase-9*, *caspase-3*, *and p53 and cytochrome c*) showed up-regulation, while (*Bcl-2*) showed down-regulation in AFB_1_ fed group. The supplementation of PCs to AFB_1_ diet significantly reversed the mRNA and protein expression of these apoptosis-associated genes, as compared to the AFB_1_ group. Our results demonstrated that PCs ameliorated AFB_1_-induced oxidative stress by modulating the antioxidant defense system and apoptosis in the BF through mitochondrial pathway in broilers.

## 1. Introduction

Aflatoxins are secondary fungal metabolites, mainly produced by *Aspergillus flavus* and *Aspergillus parasiticus*, regarded as detrimental effects to the health of animals and humans [1]. Aflatoxin B_1_ (AFB_1_) is well-known among the different aflatoxin types because of its widespread occurrence, high toxicity, and economic implications across the world [2]. According to the directions of the International Agency for Research on Cancer (IARC), AFB_1_ is a group 1 carcinogen [3]. AFB_1_ is well known for its hepatotoxic, carcinogenic, teratogenic, immunosuppressive, and other devastating effects in mammals and poultry [4,5,6,7]. Furthermore, oxidative stress has been stated to have an essential role in the AFB_1_ toxicity mechanism [8]. AFB_1_ induces the formation of free radicals, hence raising the oxidative damage and lipid peroxidation which, in turn, leads to massive cellular damage which ultimately causes death to animals and humans [9,10].

Apoptosis is the phenomenon of programmed cell death [11]. It happened in multicellular organisms. Apoptosis is an essential process for normal homeostasis of tissue; also it is associated with causing pathogenesis of various diseases [12]. Apoptosis mechanism has been experimentally tested in multiple models that aflatoxins induce apoptosis via cellular toxicity, inhibition of carbohydrate, lipid metabolism, and protein synthesis [13]. In poultry, AFB_1_ can severely affect the immune system, caused oxidative stress, apoptosis, and histopathological lesions in lymphoid tissues [14,15,16]. At the same time, AFB_1_ can change the size of the immune organs, hence severely altering the immunological functions in chickens [14,17].

Recently, chemopreventive agents derived from natural plants are getting more attention for their low toxicity and efficient therapeutic uses. One among such phytochemical agent used in toxicity experiments is proanthocyanidins, mainly derived from the grape seed extract. Proanthocyanidins (PCs) demonstrates a broad range of biological effects, including anti-carcinogenic, anti-inflammatory, anti-arthritic, anti-apoptotic, anti-mutagenic, neuroprotective, and anti-allergic properties [18,19,20,21,22,23]. 

The bursa of Fabricius (BF) is a central lymphoid organ in birds, having the main role in the establishment and maintenance of humoral immunity and B cells compartment [24]. Although, a previous study showed that AFB_1_ could inhibit the development of BF [14]. However, the therapeutic effects of PCs against AFB_1_-induced toxic effects on BF of broilers have been rarely studied, especially oxidative stress and apoptosis. Therefore, this prompted us to investigate the protective effects of PCs toward AFB_1_-induced toxic effects on the BF of broilers. The objective of the current study was to evaluate the ameliorative effects of PCs against AFB_1_-induced histopathology, oxidative stress, and apoptosis via mitochondrial-mediated apoptosis pathway in the BF of broilers. These findings will be helpful for the dietary use of PCs as a therapeutic agent against AFB_1_-induced toxicity in both human and animals.

## 2. Results

### 2.1. The Relative Weight of Bursa of Fabricius 

**Figure 1:**
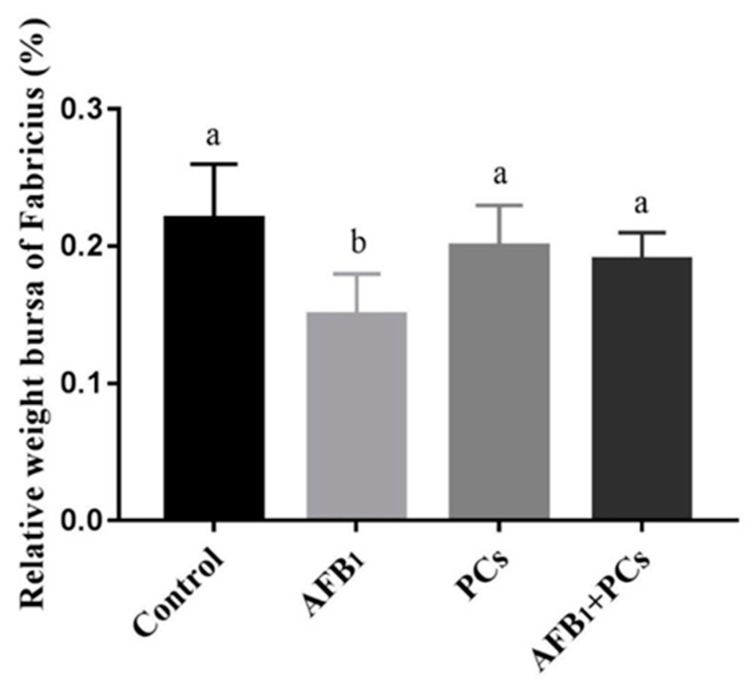
Effect of proanthocyanidins (PCs) on the relative weight of bursa of Fabricius (BF) in control and experimental broilers exposed to Aflatoxin B_1_ (AFB_1_). All data were expressed as mean ± SD (*n* = 6). Columns with different letters (a,b) indicate a significant difference at (*p* < 0.05).

As shown in Figure 1, the relative weight of bursa of Fabricius (BF) in the AFB_1_ treated group was significantly lowered in contrast with the control group. Compared to the AFB_1_ group the relative weight of BF in the AFB_1_+ PCs group was (*p* < 0.05) increased.

### 2.2. Pathological Observation

**Figure 2:**
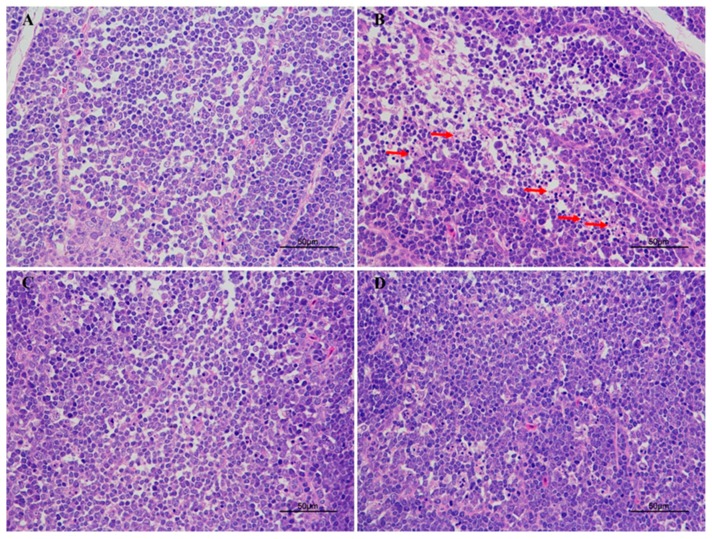
Effect of PCs on AFB_1_-induced histopathology in the control and experimental broilers. (**A**) The BF tissue from the control group; (**B**) the BF tissue, challenged with AFB_1_, induced apoptotic cells (arrows); (**C**) BF tissue from group of broilers treated with PCs; and (**D**) BF tissue from the group of broilers challenged with AFB_1_ and treated with PCs showing amelioration. The BF sections were stained with hematoxylin and eosin.

Histological changes in broiler BF tissues were shown in Figure 2. There were no pathological alterations in control and PCs alone fed groups (Figure 2A,C). The BF from AFB_1_ fed group birds manifested apoptotic cells, as compared to control and experimental groups (Figure 2B). Contrastingly, the supplementation of PCs to AFB_1_ contaminated diet significantly ameliorated and restored AFB_1_-induced apoptotic cells in the BF as compared to the AFB_1_-challenged group (Figure 2D).

### 2.3. Determination of Oxidative Stress Markers 

**Figure 3:**
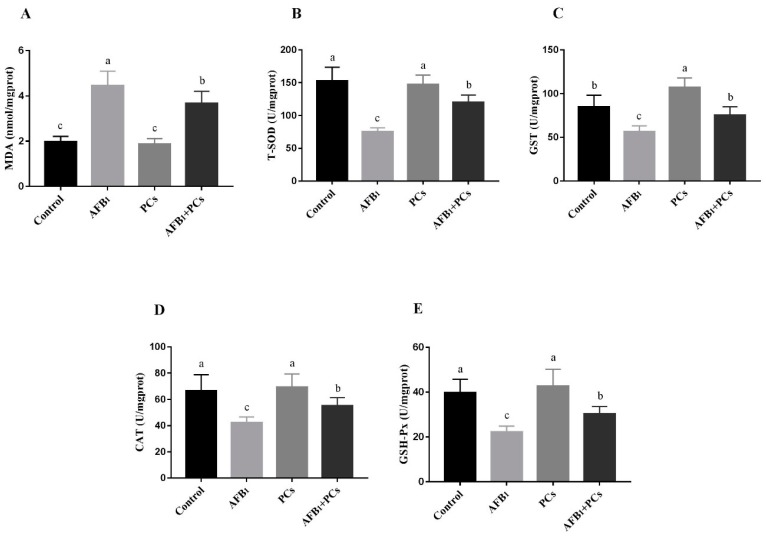
Effect of PCs on AFB_1_-induced oxidative stress markers in the control and experimental broilers. All data were expressed as mean ± SD (*n* = 6). Columns with different letters (a–c) indicate a significant difference at *p* < 0.05. (**A**) Malondialdehyde (MDA); (**B**) Total superoxide dismutase (T-SOD); (**C**) Glutathione S-transferase (GST). (**D**) Catalase (CAT); (**E**) Glutathione peroxidase (GSH-Px).

To examine the lipid peroxidation and antioxidant capacity in the BF of experimental broilers, the activities of total superoxide dismutase (T-SOD), glutathione S-transferase (GST), catalase (CAT), and glutathione peroxidase (GSH-Px) and contents of malondialdehyde (MDA) were measured (Figure 3). AFB_1_ group showed a significant increased MDA content in BF, as compared with the control group. In contrast, compared to the AFB_1_ group the MDA content was (*p* < 0.05) decreased in the group treated with AFB_1_ and PCs. Furthermore, the activities of T-SOD, GST, CAT, and GSH-Px were significantly decreased in AFB_1_ fed group when compared to the control group. However, the supplementation of PCs to AFB_1_ diet resulted in a significant improvement in the activity of T-SOD, GST, CAT, and GSH-Px when compared to the AFB_1_ fed group. In addition, PCs alone treated group exhibited a (*p* < 0.05) increase in the activity of GST in comparison with the control group (Figure 3C).

### 2.4. Relative mRNA Expression of Antioxidant-Related Genes 

**Figure 4:**
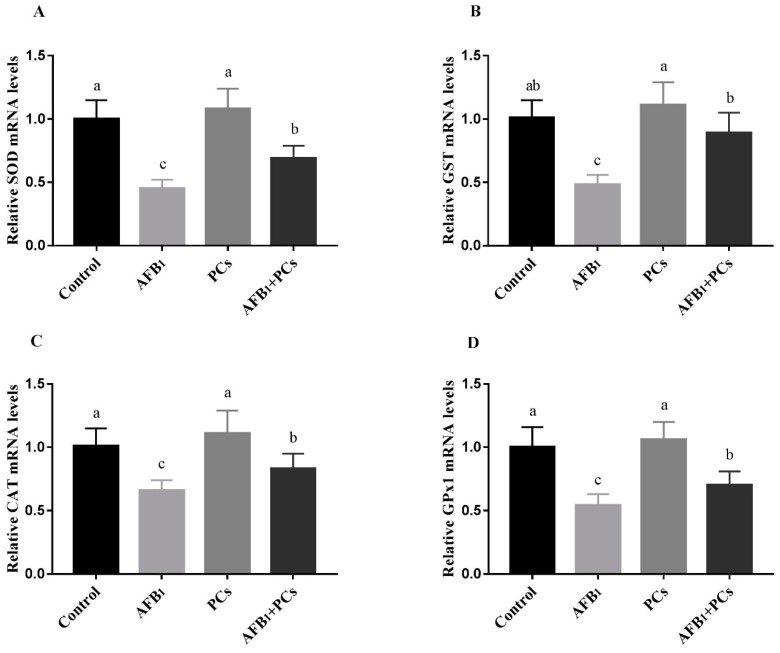
Effect of PCs on AFB_1_-induced oxidative stress-related genes in the control and experimental broilers. All data were expressed as mean ± SD (*n* = 6). Columns with different letters (a–c) indicate a significant difference at *p* < 0.05. (**A**) Superoxide dismutase (*SOD*); (**B**) Glutathione S-transferase (*GST*); (**C**) Catalase (*CAT*); (**D**) Glutathione peroxidase 1 (*GPx1*).

The messenger RNA (mRNA) expression of antioxidant genes *SOD*, *GST*, *CAT*, and *GPx1* in BF of broilers are shown in Figure 4. The results of quantitative real-time PCR showed that the mRNA expression levels of *SOD*, *GST*, *CAT*, and *GPx1* were (*p* < 0.05) down-regulated in the AFB_1_ group, compared to the control group. However, the addition of PCs into the AFB_1_ contaminated diet resulted in a significant up-regulation on the relative mRNA levels of *SOD*, *GST*, *CAT*, and *GPx1*, when compared to the AFB_1_ group. 

### 2.5. Relative mRNA Expression Levels of Apoptosis-Associated Genes 

**Figure 5:**
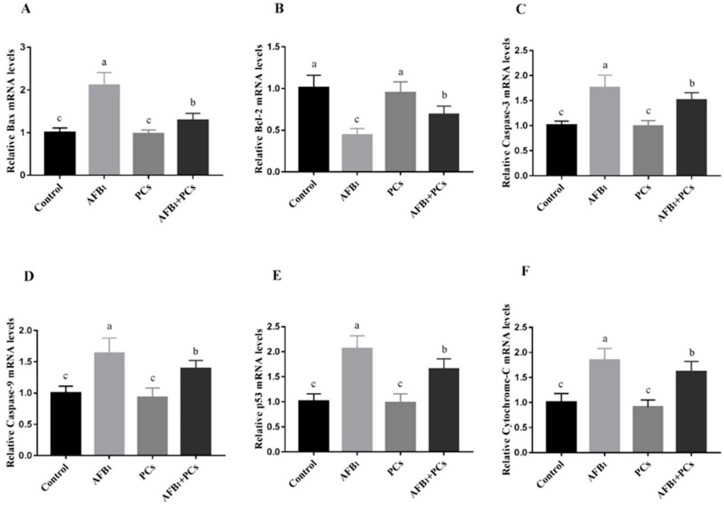
Effect of PCs on mRNA levels of mitochondrial apoptosis-associated genes in the control and experimental broilers exposed to AFB_1_. All data were expressed as mean ± SD (*n* = 6). Columns with different letters (a–c) indicate a significant difference at (*p* < 0.05). (**A**) Bcl-2-associated X protein (*Bax*); (**B**) B-cell lymphoma 2 (*Bcl-2*); (**C**) *caspase-3*; (**D**) *caspase-9*; (**E**) tumor protein p53 (*p53*); and (**F**) *cytochrome c*.

The effects of AFB_1_ and/or PCs on the mRNA expression levels of *Bax*, *Bcl-2*, *caspase-3*, *caspase-9*, *p53*, and *cytochrome-C* in control and experimental groups are depicted in Figure 5**.** Compared to the control group, the mRNA expression of *Bax*, *caspase-3*, *caspase-9*, *p53*, and *cytochrome-C* was significantly up-regulated in the AFB_1_ treated group. However, the supplementation of PCs to AFB_1_ diet (*p* < 0.05) reversed AFB_1_-induced up-regulation in the mRNA expression of *Bax*, *caspase-3*, *caspase-9*, *p53*, and *cytochrome-C*, as compared with the AFB_1_ group. The *Bcl-2* mRNA expression level markedly down-regulated in the AFB_1_ fed group, when compared to the control group (Figure 5B). In contrast, compared to the AFB_1_ treated group, there was significant up-regulation in the *Bcl-2* mRNA expression in the group co-treated with AFB_1_ + PCs.

### 2.6. Protein Expression Levels of Apoptosis-Associated Genes 

**Figure 6:**
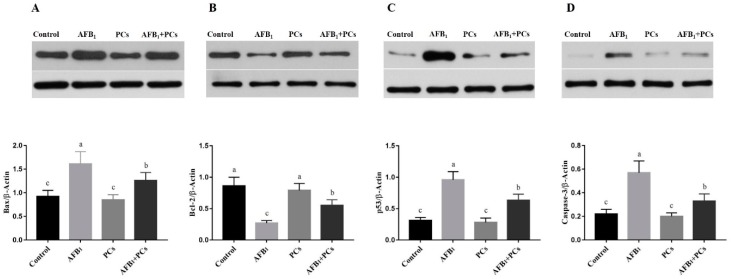
Effect of PCs on protein expression levels of mitochondrial apoptosis-associated genes in the control and experimental broilers. All data were expressed as mean ± SD. Columns with different letters (a–c) indicate a significant difference at (*p* < 0.05). The lower bands are representing β-Actin for all above validated proteins. (**A**) Bcl-2-associated X protein (Bax); (**B**) B-cell lymphoma 2 (Bcl-2); (**C)** tumor protein p53 (p53); and (**D**) caspase-3.

To explore the protective effects of PCs on AFB_1_-induced apoptosis, we further determined the protein expression level of Bax, Bcl-2, p53, and caspase-3 in broiler BF by Western blotting (Figure 6). Interestingly, the same trends were noted in Bax, Bcl-2, p53, and caspase-3 protein expressions as indicated in the mRNA expression levels. AFB_1_ treated group showed significant up-regulation in the protein expression of Bax, p53, and caspase-3 (Figure 6A,C,D). In contrast (*p* < 0.05) down-regulation was observed in the protein expression of Bcl-2 as compared to the control group (Figure 6B). However, compared to the AFB_1_ group, the significant down-regulation of Bax, p53, and caspase-3, and (*p* < 0.05) up-regulation in Bcl-2 were noticed in the group treated with AFB_1_ and PCs.

## 3. Discussion

Proanthocyanidins are natural antioxidants with a wide range of pharmacological and medicinal properties. The absorption of PCs initially takes place in the small intestine and, subsequently, liver, thus resulting in a wide range of metabolites that reach to other organs through bloodstream [25]. In poultry the BF is a primary lymphoid organ which is responsible for the proliferation and modification of B cells [26]. Its relative weight usually evaluates the developmental status of BF. In our current study, AFB_1_ treated group reduced the relative weight of the BF, as compared with the control group. Moreover, our results showed that broilers challenged with AFB_1_ resulted in apoptotic cells in the BF, these findings were consistent with previous reports [14,27]. Strikingly, the supplementation of PCs to AFB_1_ contaminated diet ameliorated and restored AFB_1_-induced apoptotic cells in the BF as compared to the AFB_1_ challenged group and significantly improved the relative weight of BF altered by AFB_1_.

Aflatoxins have been reported to trigger the production of reactive oxygen species (ROS) and weaken antioxidant defense system, thus resulting in deoxyribonucleic acid (DNA) and mitochondrial damages, and the induction of apoptosis [28,29]. The cellular damage and lipid peroxidation status can be recognized by measuring the MDA content, as MDA is the main product of polyunsaturated lipid peroxidation [30]. The GST, SOD, GSH-Px, and CAT which are the most important elements of the endogenous antioxidant defense system, play a key role in relieving oxidative damage and free radicals scavenging and maintain the intracellular redox balance [31]. These antioxidant enzymes have been validated to have a critical role in the antioxidant mechanism of the body that can eliminate ROS from the cell, such as, for the first line of defense SOD catalyzes the dismutation of superoxide anion (O_2_^−^) to hydrogen peroxide (H_2_O_2_) [32]. CAT, a primary antioxidant defense component, catalyzes H_2_O_2_ directly to H_2_O and O_2_ with the presence of GSH. GSH-Px helps to metabolize H_2_O_2_ to non-toxic products and halts lipid peroxidation [33,34]. In the present study, the T-SOD, GST, CAT, and GSH-Px, activities as well as the MDA contents were also measured to assess the oxidative status of BF. Our results indicated that T-SOD, GST, CAT, and GSH-Px, activities were significantly decreased, while MDA content markedly increased in the AFB_1_ fed group, as compared to the control group. The current study findings are in accordance with the previous studies which revealed that AFB_1_ could induce oxidative stress in broilers [5,15,35,36]. Furthermore, PCs reduced the MDA contents and markedly increased the antioxidant enzyme activities (T-SOD, GST, CAT, and GSH-Px,), when compared to the AFB_1_ group. The above results were further confirmed by the mRNA expression analysis of antioxidant genes. Notably, in our study PCs enhanced GST enzyme activity and gene expression which is crucial for the detoxification of AFB_1_ and its toxic metabolites [37]. The present study findings confirmed that PCs counteracted oxidative stress and enhanced the antioxidant defense system induced by AFB_1_ in the BF of broilers.

Apoptosis is the phenomenon of programmed cell death that may occur in multicellular organisms [38]. Mitochondrial-dependent apoptotic signaling pathway plays a crucial role in the process of apoptosis. It is well documented that members of the Bcl-2 proteins regulate the mitochondrial-dependent apoptotic pathway including both anti-apoptotic (Bcl-2), and pro-apoptotic (Bax) [39]. Bcl-2 is one of anti-apoptotic protein which protects cells from apoptosis and pro-apoptotic proteins such as Bax that stimulate cell death [40]. Moreover, p53 protein can trigger the transcriptional activation of apoptotic factors such as Bax. Additionally, it can be transferred to the mitochondria, then binds to the Bcl-2 protein, thereby counteracting the anti-apoptotic role of Bcl-2 [41]. The caspase family has an essential function in the apoptosis pathway, and caspase-3 plays a key role in mediating in the mitochondrial pathway [42]. Throughout the transduction of an apoptotic (death) signal into the cell, an alteration occurs in the permeability of the membranes of the mitochondrial cells. As a consequence translocation of the apoptogenic proteins such as cytochrome complex (Cyt c) into the cytoplasm, that activates the initiation of caspase-9 and leads to the caspase-3 activation, thus induction of apoptosis [43,44]. Furthermore, previous studies reported that aflatoxins react antagonistically with different cell proteins, leading to inhibition of carbohydrate and lipid metabolism and protein synthesis, which induces apoptosis [13]. In this study, we determined the expression patterns of apoptosis genes involved in the mitochondrial pathway and therapeutic effects of PCs in preventing AFB_1_-induced apoptosis in BF of broilers. Our results showed that the mRNA expression levels of *Bax*, *caspase-3*, *caspase-9*, *p53*, and *cytochrome-C* were markedly increased while the mRNA expression levels of *Bcl-2* were significantly decreased in the AFB_1_-fed group. Additionally, the same trends were observed in the protein expression profile of Bax, Bcl-2 caspase-3, and p53. The Western blot results showed that exposure to AFB_1_ significant up-regulation was noted in the protein expression of Bax, caspase-3, and p53, whereas down-regulation in the Bcl-2, when compared to the control and PCs alone treated groups. Similar results were noted in previous studies. Researchers reported that AFB_1_ exposure could result in apoptosis in the liver [45], BF [14], spleen [15], and thymus [46] of broilers, the mechanism associated with the mitochondrial pathway. However, the inclusion of PCs into AFB_1_ contaminated diet prevented AFB_1_-induced up-regulation in *Bax*, *caspase-3*, *caspase-9*, *p53*, and *cytochrome-C* and down-regulation in *Bcl-2* mRNA expression levels as compared to the AFB_1_ treated group. Moreover, the supplementation of PCs along with an AFB_1_-contaminated diet reversed AFB_1_-induced protein expressions of Bax, Bcl-2, caspase-3, and p53 when compared with the AFB_1_ group. From these results, it has been suggested that PCs prevented AFB_1_-induced apoptosis in the BF of broilers by modulating both mRNA and protein expression levels of apoptosis-associated genes through the mitochondrial pathway. 

## 4. Conclusions

In conclusion, our results suggested that AFB_1_ exerted toxic effects in the BF of broilers, as it induced oxidative stress and apoptosis. Furthermore, histology results showed that PCs ameliorated AFB_1_-induced apoptotic cells. In addition, PCs inhibited AFB_1_-induced oxidative stress by reducing the LPO accumulation and enhancing the antioxidant enzymes capacity. Notably, PCs attenuate AFB_1_-induced excessive apoptosis through the mitochondrial-mediated apoptosis pathway in the BF of broilers. The current study findings will provide helpful insight into the dietary use of PCs as a therapeutic agent against AFB_1_-induced toxicity in both humans and animals.

## 5. Material and Methods 

### 5.1. Animal Ethics

All the experimental design and protocols were approved by the Institutional Animal Care and Ethics Committee of Huazhong Agricultural University, Wuhan, China on 7 August 2017 (approval no. HZAUCH-2017-007). 

### 5.2. Aflatoxin B_1_ Production and Analysis

The *Aspergillus flavus* strain (NRRL-3357) was obtained from Sun Yat-sun University China. The strain was sub-cultured on potato dextrose agar (E. Merck, Darmstadt, Germany) medium at 30 °C for seven days. AFB_1_ was produced on corn following the method according to our previous study [47]. After inoculation of corn it was incubated for 15 days to get the approximate AFB_1_ content of 64 mg/kg. The concentration of AFB_1_ was determined by high-performance liquid chromatography (HPLC) (Waldbronn, Germany) equipped with fluorescent detector. Chromatographic separation was done using a C_18_ column (250 × 4.6 mm, 5 µm, Agilent Technologies, Santa Clara, CA, USA) as previously described [47].

### 5.3. Bird, Diets, and Management

Proanthocyanidins, extracted from grape seed were obtained from Zelang Medical Technology Company (Nanjing, China; purity *≥* 98%). One hundred forty-four one-day old Cobb broilers were purchased from a commercial hatchery (Jingzhou Kang Poultry Co., Ltd., Jingzhou, China). To ensure the proper distribution of artificially AFB_1_ contaminated feed and PCs in the basal diet. The basal diet was mixed with AFB_1_ contaminated feed and PCs in Rotex Master Mixer model YSHJ-100 (Shandong Rotex Machinery Co; Ltd, Shandong, China) for 20 min. After acclimatization for three days all the birds were randomly allocated into four groups with six replicates; six birds each replicate (*n* = 36 per group). Groups were distributed based on the following four dietary treatments: (1) basal diet without addition of PCs or AFB_1_ (Control); (2) basal diet supplemented with 1 mg/kg AFB_1_ from contaminated corn (AFB_1_); (3) basal diet supplemented with 250 mg/kg PCs (PCs); and (4) basal diet supplemented with 1 mg/kg AFB_1_ + 250 mg/kg PCs (AFB_1_ + PCs). Birds were reared in cages with electrically-heated units and feed and water were provided ad libitum throughout the experimental period (28 days). The experiment was conducted under controlled environmental conditions. The composition of the basal diet has been presented in Table 1.

### 5.4. Collection of Samples

At the 28 days of age, one bird randomly selected from each cage (six birds from each group). Birds were individually weighed, and euthanized by cervical dislocation, and then bursa of Fabricius (BF) was collected and weighed immediately. The BF was immersed in liquid nitrogen and preserved at −80 °C for further analysis. The relative weight of each collected BF was calculated by the following formula:

Relative weight = organ weight (g)/body weight (kg)

### 5.5. Pathological Observation

For histological examination, the BF tissues were fixed in 10% neutral formalin. The hematoxylin and eosin (H and E) staining were performed according to previously described [35]. The BF sections of all birds were microscopically examined.

### 5.6. Determination of Oxidative Stress

The BF samples for determination of oxidative stress were prepared as described in our previous study [35]. Briefly, BF tissue samples (0.5 g) were cut into small pieces and homogenized in 4.5 mL ice cold physiological saline. The homogenate was centrifuged at 1000× *g* for 15 minutes at 4 °C. The supernatant was collected and stored at −80 °C for the following analysis. The contents of malondialdehyde (MDA) and activities of catalase (CAT), glutathione peroxide (GSH-Px), glutathione-S transferase (GST) and total superoxide dismutase (T-SOD), in the BF supernatants were measured spectrophotometrically (Hengping, Shanghai, China) using assay kits (Nanjing Jiancheng Bioengineering Institute, Nanjing, China). The details of all the determination procedures were performed according to the manufacturer’s protocols.

### 5.7. Total RNA Extraction and Quantitative Real-Time PCR

Total mRNA from the tissues of BF was extracted with Trizol^®^ (Invitrogen, Carlsbad, CA, USA) following the manufacturer’s instructions. The quality and concentration of RNA samples were measured by nucleic acid concentration analyzer NanoDrop 2000 (Thermo Fisher, Waltham, MA, USA). The complementary DNA (cDNA) was obtained from 1 µg of total ribonucleic acid (RNA) through reverse transcription in a 20 µL mixture reaction using a PrimeScript^TM^ RT reagent Kit (Takara DRR037A, Dalian, China) following the manufacturer’s protocol. The expression levels of pertaining genes (*β-actin*, *SOD*, *CAT*, *GPx1*, *GST*, *Bax*, *Bcl-2*, *caspase-9*, *caspase-3*, *p53*, and *cytochrome-c*) were quantified by quantitative real-time PCR (CFX384, Bio-Rad, Hercules, CA, USA) using the SYBER® Green PCR Master Mix (Applied Biosynthesis, Waltham, UK), following the method according to our previous study [48], and the manufacturer’s instructions. The primer sequences of each gene used in this study are listed in Table 2. The 2^−△△*C*t^ method was used for quantification with the β-actin as a reference gene, and the relative abundance was normalized to the control (as 1) [49,50]. The findings were expressed as relative mRNA levels.

### 5.8. Western Blot Analysis

Protein expressions of Bax, Bcl-2, caspase-3, and p53 in the BF of broilers were estimated by western blot according to our previous study [51]. The following antibodies used for the present study were purchased from indicated sources: Anti-Bax, Bcl-2, caspase-3, and p53 (Abclonal Technology, Wuhan, China), and Anti-β-Actin, (Cell Signaling Technology, Boston, MA, USA). The HRP-labeled goat anti-rabbit IgG (Servicebio Technology, Wuhan, China) was used as the secondary antibody. Samples were analyzed in triplicate; a representative blot is shown in respective figures. Proteins bands were detected through chemiluminescence WesternBright^TM^ ECL substrate kit (Advansta, Menlo Park, CA, USA), then visualized and quantified by a FluroChem FC2 Imaging System (ProteinSimple, California, USA).

### 5.9. Statistical Analysis

The experimental data were analyzed by one-way ANOVA using IBM SPSS Statistic 22 (IBM Corporation, Armonk, NY, USA). Differences were considered to be significant at *p* < 0.05, the Duncan’s test was used to separate the significant differences between means. Results were presented as mean ± SD.

## Figures and Tables

**Table 1:** Basal diet formulation and nutritional value.

Ingredient	(%)
Corn	58.3
Soybean meal	30.2
Fish meal	5.6
Soybean oil	2.3
Dicalcium phosphate	1.2
Lime stone	1.00
Salt	0.2
Methionine	0.2
Premix ^1^	1.00
Total	100.00
**Calculated chemical composition**	
Crude protein	21.87
Metabolisable energy (MJ/kg)	13.45
Lysine	1.14
Methionine	0.40
Methionine + Cystine	0.94
Calcium	0.95
Available phosphorus	0.49

^1^ The premix contained (per kg of diet): Fe, 60 mg; Cu, 7.5 mg; Zn, 65 mg; Mn, 110 mg; I, 1.1 mg; Se, 0.4 mg; biotin, 0.04 mg; choline chloride, 400 mg; vitamin A (from retinyl acetate), 4500 IU; vitamin D3 (from cholecalciferol), 1000 IU; vitamin K (menadione sodium bisulphate), 1.3 mg; vitamin B1, 2.2 mg; vitamin B2, 10 mg; vitamin B3, 10 mg; vitamin B5, 50 mg; vitamin B6, 4 mg; vitamin B11, 1 mg; vitamin B12, 0.013 mg.

**Table 2:** Primers used for quantitative real-time PCR.

Target Gene	Primer	Primer Sequence (5′*→*3′)	Accession No.
β-Actin	Forward Reverse	CCCGCAAATGCTCTAAACC CCAATCCTGTCTTGTTTTATGC	L08165
Bax	Forward Reverse	TCCTCATCGCCATGCTCAT CCTTGGTCTGGAAGCAGAAGA	XM_422067
Bcl-2	Forward Reverse	CGCCGCTACCAGAGGGACTT CCGGACCCAGTTGACCCCAT	Z_11961.1
Caspase-9	Forward Reverse	CCAACCTGAGAGTGAGCGATT GTACACCAGTCTGTGGGTCGG	AY057940
Caspase-3	Forward Reverse	GGCTCCTGGTTTATTCAGTCTC ATTCTGCCACTCTGCGATTT	NM_204725.1
p53	Forward Reverse	GCCGTGGCCGTCTATAAGAA GGTCTCGTCGTCGTGGTAAC	NM_205264.1
SOD	Forward Reverse	CGTCATTCACTTCGAGCAGAAGG GTCTGAGACTCAGACCACATA	NM_205064
GPx1	Forward Reverse	GACCAACCCGCAGTACATCA GAGGTGCGGGCTTTCCTTTA	NM_001277853.1
CAT	Forward Reverse	CCACGTGGACCTCTTCTTGT AAACACTTTCGCCTTGCAGT	NM_001031215.1
GST	Forward Reverse	AGTCGAAGCCTGATGCACTT TCTAGGCGTGGTTTCCTTTG	L15386.1
Cytochrome-C	Forward Reverse	CGCAGGCTCCATACTACTCG TTAGGGCACCTCATAGGGCT	NC_001323.1
